# Lipid-Based Nanoparticle Formulation of Diallyl Trisulfide Chemosensitizes the Growth Inhibitory Activity of Doxorubicin in Colorectal Cancer Model: A Novel In Vitro, In Vivo and In Silico Analysis

**DOI:** 10.3390/molecules27072192

**Published:** 2022-03-28

**Authors:** Faris Alrumaihi, Masood Alam Khan, Ali Yousif Babiker, Mohammed Alsaweed, Faizul Azam, Khaled S. Allemailem, Ahmad A. Almatroudi, Syed Rizwan Ahamad, Mahdi H. Alsugoor, Khloud Nawaf Alharbi, Nahlah Makki Almansour, Arif Khan

**Affiliations:** 1 Department of Medical Laboratories, College of Applied Medical Sciences, Qassim University, Buraydah 51452, Saudi Arabia; f_alrumaihi@qu.edu.sa (F.A.); ababkr@qu.edu.sa (A.Y.B.); k.allemailem@qu.edu.sa (K.S.A.); aamtrody@qu.edu.sa (A.A.A.); khloud.n.alharbi@gmail.com (K.N.A.); 2 Department of Basic Health Sciences, College of Applied Medical Sciences, Qassim University, Buraydah 51452, Saudi Arabia; a_khan@qu.edu.sa; 3 Department of Medical Laboratory Sciences, College of Applied Medical Sciences, Majmaah University, Al-Majmaah 11952, Saudi Arabia; m.alsaweed@mu.edu.sa; 4 Department of Pharmaceutical Chemistry and Pharmacognosy, Unaizah College of Pharmacy, Qassim University, Unaizah 51911, Saudi Arabia; f.azam@qu.edu.sa; 5 Department of Pharmaceutical Chemistry, College of Pharmacy, King Saud University, Riyadh 11451, Saudi Arabia; srahamad@ksu.edu.sa; 6 Department of Emergency Medical Services, Faculty of Health Sciences, AlQunfudah, Umm Al-Qura University, Makkah 21912, Saudi Arabia; mhsugoor@uqu.edu.sa; 7 Department of Biology, College of Science, University of Hafr Al Batin, Hafr Al Batin 31991, Saudi Arabia; nahlama@uhb.edu.sa

**Keywords:** diallyl trisulfide stealth liposomes, doxorubicin PEGylated liposomes, chemoprevention, chemosensitization, colorectal cancer, molecular docking

## Abstract

Garlic’s main bioactive organosulfur component, diallyl trisulfide (DATS), has been widely investigated in cancer models. However, DATS is not suitable for clinical use due to its low solubility. The current study seeks to improve DATS bioavailability and assess its chemopreventive and chemosensitizing properties in an AOM-induced colorectal cancer model. The polyethylene glycol coated Distearoylphosphatidylcholine/Cholesterol (DSPC/Chol) comprising DATS-loaded DATSL and doxorubicin (DOXO)-encapsulated DOXL liposomes was prepared and characterized. The changes in the sensitivity of DATS and DOXO by DATSL and DOXL were evaluated in RKO and HT-29 colon cancer cells. The synergistic effect of DATSL and DOXL was studied by cell proliferation assay in the combinations of IC_10_, IC_25_, and IC_35_ of DATSL with the IC_10_ of DOXL. AOM, DATSL, and DOXL were administered to different groups of mice for a period of 21 weeks. The data exhibited ~93% and ~46% entrapment efficiency of DATSL and DOXL, respectively. The size of sham liposomes was 110.5 nm, whereas DATSL and DOXL were 135.5 nm and 169 nm, respectively. DATSL and DOXL exhibited significant sensitivity in the cell proliferation experiment, lowering their IC_50_ doses by more than 8- and 14-fold, respectively. However, the DATSL IC_10_, IC_25_, and IC_35_ showed escalating chemosensitivity, and treated the cells in combination with DOXL IC_10_. Analysis of histopathological, cancer marker enzymes, and antioxidant enzymes revealed that the high dose of DATSL pretreatment and DOXL chemotherapy is highly effective in inhibiting AOM-induced colon cancer promotion. The combination of DATSL and DOXL indicated promise as a colorectal cancer treatment in this study. Intermolecular interactions of DATS and DOXO against numerous cancer targets by molecular docking indicated MMP-9 as the most favourable target for DATS exhibiting binding energy of −4.6 kcal/mol. So far, this is the first research to demonstrate the chemopreventive as well as chemosensitizing potential of DATSL in an animal model of colorectal cancer.

## 1. Introduction

According to GLOBOCAN 2020, the incidence of colorectal cancer (CRC) has been placed in third position, following breast and lung cancer, but found second rank among global cancer-related deaths in the year 2020 [1]. As approximately 15% of new cases of all types of cancer were recorded as CRC in 2020, it has been a steadily growing concern in Saudi Arabia. However, cancer-related death was assessed to be second, as 14% of deaths were estimated from CRC, while 14.2% were recorded from breast cancer [1]. The delayed diagnosis of CRC makes therapeutic strategies more challenging due to the poor prognosis of the disease [2,3,4]. Therefore, surgical removal followed by chemotherapy is the initial treatment choice in CRC, though it is not eradicated totally [5,6,7]. The use of chemotherapeutic agents, monoclonal antibodies, radiation therapy, and surgery have shown limitations, as resistance to drugs, side effects, and cancer recurrence was recorded by these therapeutics [8,9,10,11,12]. However, the initiation, promotion, and progression of all types of cancer, including CRC, have been associated with multiple signalling pathways, weakening the treatment strategies based on a single drug or targeting a single gene [13,14,15]. Evidently, combination therapy has been shown to overcome the challenges of monotherapy, as several molecular targets can be approached. However, the selection of suitable drugs and their ratios, with minimal side effects, is another challenge in developing novel therapeutic strategies [16,17,18,19].

In recent years, numerous research supported the use of secondary metabolites as cancer chemopreventive and therapeutic agents. The epidemiological studies also demonstrated the role of dietary habits in preventing various diseases, including cancer. The data from such studies suggest that adding some foods to the diet reduces the risk of cancer and cancer-related death by more than 30% [20,21,22]. Remarkably, statistics from the drug development process reveal that half of the pharmaceuticals available commercially over the past three decades were either derived naturally or altered chemically [20,23,24,25,26].

Epidemiological studies have supported that more than 70% of CRC occurred due to the consumption of an unhealthy diet and lifestyle [27]. Since ancient times, garlic has been one of the most popular food flavouring spices and used traditionally as medicine to treat multiple diseases, including cancer [28,29]. Various studies supported that including allium vegetables in the diet may prevent the risk of cancer, including CRC [30,31,32]. Multiple analytical studies show that the abundance of organosulfur compounds in garlic has an active role in flavour and therapeutic efficacies. Several organosulfur compounds have been shown to inhibit all the stages of carcinogenesis in different cancer models. The crushing of garlic converts S-allylcysteine sulfoxide into dially thiosulfonate, which is mainly transformed to oil-soluble diallyl sulfide (DAS), diallyl disulfide, and dially trisulfide (DATS) [33,34,35]. These allyl sulfides have been shown to induce apoptosis in different cancer cells through multiple signalling pathways [36,37,38,39]. Interestingly, DATS has been consumed frequently in China as a supplement, and synthesized commercially at a broad level [40]. Several studies have demonstrated the anticancer potential of DATS by inhibiting the cellular proliferation, migration, and invasion in various cancer cells and in chemically induced animal models [41,42,43]. DATS also showed the induction of a mitochondria-dependent apoptotic pathway by the upregulation of cytochrome C and caspase-3 and -9, in human primary CRC cells [44]. The treatment of DATS reduced the expression of PI3K, Ras, and p38 in colo 205 colon cancer cells [45].

However, regardless of high potential, the major drawback of DATS is its very low solubility in water, which confines its wide-ranging uses clinically. Thus, preparing suitable formulations of DATS can overcome this limitation to utilize its efficacy against several pathological conditions. Recently, we prepared the PEG-coated DSPC/Chol liposomes of diallyl disulfide and thymoquinone and demonstrated their stability as well as efficacy against HCT116 and RKO colon cancer and A549 and H460 lung cancer lines respectively [46,47]. The liposomal formulation of diallyl disulfide increased the efficacy oxaliplatin by inducing ROS, consequently leading to apoptosis [47]. Earlier, we also prepared the pH-sensitive liposomal formulations of diallyl sulfide and showed its chemopreventive potential in dimethyl-benz-a-anthracene (DMBA) induced skin papilloma [48]. Multiple studies have reported the role of liposomal formulation of secondary metabolites to improve the efficacy of chemotherapeutic agents [49,50]. Earlier, curcumin-loaded liposomes showed to inhibit the tumour growth inhibition in combination with doxorubicin and oxaliplatin liposomes in CRC cancer cells and tumour xenografts mice [51,52]. Evidently, multiple studies supported the advancement of stealth, small nanosized, PEGylated liposomal formulations in the lipid-based drug delivery vehicles. The addition of polyethylene glycol (PEG) in the liposomes protects it from the accessibility of enzymes due to its thick layer of water. The long-circulating stealth liposomes improve the extravasation in solid tumours due to vascular disruptions related to tumour angiogenesis. The PEGylated liposomes make no direct interaction with the target cancer cells while releasing the drug for eventual diffusion into them [53,54,55,56]. The current research focuses on preparing and characterizing the PEGylated liposomes of DATS and DOXO and evaluating their chemopreventive and chemotherapeutic potential in colorectal cancer (CRC) models in vitro well as in vivo. The present study also aimed to identify possible molecular targets of DATS and DOXO against colorectal cancer by in silico analysis.

## 2. Methods

### 2.1. Reagents

Distearoylphosphatidylcholine (DSPC), 1,2-distearoyl-*sn*-glycero-3-phosphatiylethanolamine-N-[methoxy(polyethyleneglycol)-2000] (DSPE-PEG_2000_) and cholesterol (Chol), doxorubicin, azoxymethane (AOM), and diallyl trisulfide (DATS) were purchased from Sigma-Aldrich (St. Louis, MO, USA). The adenosine deaminase (ADA), lactate dehydrogenase (LDH), superoxide dismutase (SOD) assay, catalase (CAT) assay, lipid peroxidation (MDA) assay, glutathione peroxidase 1 (GPx1) assay, gamma glutamyl transferase (GGT), 5′-nucleotidase (CD73) activity assay and cell cytotoxicity assay (CAA) kit, were procured from Abcam (Cambridge, MA, USA). Dulbecco’s modified Eagle medium (DMEM) was purchased from Santa Cruz Biotechnology Inc, Heidelberg, Germany. Foetal bovine serum (FBS), streptomycin/penicillin, amphotericin-B were procured from Life Technologies, USA. HT-29 (ATCC HTB-38) and RKO (ATCC CRL-2577) were commercially purchased ATCC (American Type Culture Collection), Manassas, VA, USA.

### 2.2. Preparation of DATS and DOXO Entrapped Liposomes

The PEG-coated liposomal formulations of DATS and doxorubicin (DOXO) were prepared separately using DSPC/Chol/mPEG-DSPE as described earlier [46]. Briefly, the 7:3 molar ratio of DSPC:Chol were mixed with 5% and 1% mPEG-DSPE and DATS/DOXO, respectively, in the solvents, and thin lipid films were prepared in a round-bottom flask using a rotary evaporator in a N_2_ environment. The DSPC, Chol, mPEG-DSPE, and DATS were dissolved in the chloroform, and DOXO in the PBS. The unilamellar vesicles (ULVs) were achieved by the sonication of multilamellar vesicles (MLVs), following hydration of lipid film with the phosphate saline buffer. The ULVs were then passed through the decreasing pore sized polycarbonate membranes from 400–100 nm several times with the help of a handheld extruder at ambient temperature. Then, the supernatants were decanted to remove any unentrapped DATS and DOXO after centrifuging the liposomal solutions for 25 min at 25,000 rpm.

### 2.3. Characterization of Liposomes

#### 2.3.1. The Mean Particle Size, ζ Potential Polydispersity Index (PDI) and Entrapment Efficiency (EE) of DATS/DOXO Loaded and Sham Liposomes

The UV spectrophotometer was used to evaluate the entrapment efficiency (EE) of DATS as described earlier [46,47]. The liposomes were lysed with 0.5% Triton-X-100 and the amount of DATS entrapped in the liposomes was calculated by applying the formula.

$$\%\;\mathrm{Entrapment}\;\mathrm{Efficiency}\;\left(\mathrm{EE}\right)\mathrm{of}\;\mathrm{the}\;\mathrm{drug}=\frac{\mathrm{Liposome}\;\mathrm{entrapped}\;\mathrm{drug}}{\mathrm{Total}\;\mathrm{drug}}\times 100$$

The size of the DATS/DOXO-loaded and empty liposomes were evaluated in PBS, while the zeta potential as well as PDI of these preparations were analysed in the deionized water by dynamic light scattering (DLS) in Zetasizer Nano system (Malvern Instruments, Malvern, Worcestshire, UK) as described earlier [46,47].

#### 2.3.2. In Vitro Stability of DATS and DOXO Containing Liposomes and Its Release Kinetics

The stability of DATSL and DOXL was evaluated in the PBS at 37 °C by incubating the respective liposomal formulations from 1–72 h, as reported previously [46,47]. In brief, 1 mL of DATSL/DOXL was incubated in the dialysis bags (MWCO 3.5 kDA), for 72 h against 20 mL of 9% sucrose solution in PBS with continuous gentle shaking. At specific time points of 1, 2, 4, 8, 12, 18, 24, 36, 48, 60, and 72 h, 1 mL of solution was withdrawn and substituted with 1 mL of PBS. After determining the quantity of DATS at 265 nm and DOXO at 490 nm wave lengths using a UV spectrophotometer, the release of DATSL/DOXL in the PBS was estimated using the following formula, as described earlier [46,47].

The release kinetics of DATS and DOXO release DATSL and DOXL in 90% bovine serum were determined at several time points ranging from 1–72 h, as previously described [46,47]. Following incubation at specific time points, the mixture was withdrawn and centrifuged. The amount of DATS and DOXO in DATSL and DOXL, respectively, was estimated as mentioned above.

$$\mathrm{Drug}\;\mathrm{release}\;\left(\%\right)=\frac{CnV+\sum_{i=0}^{n}CiVi}{w}\times 100\%$$

*Cn* and *Ci* are the amounts of drug in the mixture at the ‘n’ and ‘I’ sampling points, respectively, while *V* and *Vi* are the final mixture (20 mL) and collected sample (1 mL) at each point, correspondingly.

### 2.4. Cell Cytotoxicity Assay

The cytotoxic effects of DATS and DOXO and their formulations (DATSL and DOXL) were evaluated by cell cytotoxicity assay in RKO and HT-29 colorectal cancer cells with a broad range of concentrations. An amount of 10^4^ cells/well in 96 wells cell culture plates were split for 24 h, followed by the treatment of cells in the concentrations given below in Table 1, for 24 h, 48 h, and 72 h in 5% CO_2_ atmosphere at 37 °C. The cell cytotoxicity reagent was added in each well following the instructions provided in the kit. The viability of the cells was determined by the formula below, after measuring the absorbance at 590 nm. Further, the IC_10_, IC_25_, and IC_35_ for DATSL and IC_10_ for DOXL were selected to assess the potential of DATSL and DOXL in various combinations.

(1) $$\%\;\mathrm{Cell}\;\mathrm{Viability}=100\times \frac{\left(A_{sample}-A_{0}\right)}{\left(A_{Ctrl}-A_{0}\right)}$$

where *A_sample_* is the absorbance recorded DATS/DATSL/DOXO/DOXL exposed cells; *A_ctrl_* is the absorbance of vehicle exposed cells; *A*_0_ is the absorbance of media without cells.

### 2.5. In Vivo Studies

#### 2.5.1. Mice

Male Swiss albino mice with aged of 8 to 10 weeks were purchased from the animal facility in the KSU, Riyadh, Saudi Arabia. All animal experiments, including the induction of lung cancer using chemical carcinogen, bleeding, and injection, and the sacrifice of the mice were conducted following guidelines of the University of London Animal Welfare Society, Wheathampstead, England. The study protocol was approved by Animal Ethical Committee, Qassim University, Saudi Arabia as cams1-2019-2-2-I-5645. All the experimental animals were housed in the animal house facility of CAMS under specific pathogen-free conditions at a controlled temperature of 23 ± 2 °C, relative humidity 55 ± 10%, and with 12 h light/12 h dark cycles. They were given food and water ad libitum and their health was checked during the entire experimental period at least twice a day by well-trained and dedicated staff members. All the surviving animals were normally euthanized by CO_2_ inhalation at the end of the study. However, the mice were also euthanized in a CO_2_ chamber within 2–4 h if they were moribund, measured by a lack of sustained purposeful response to gentle stimuli. None of the mice died during the experiment before euthanasia.

#### 2.5.2. Experimental Design

**Figure 1:**
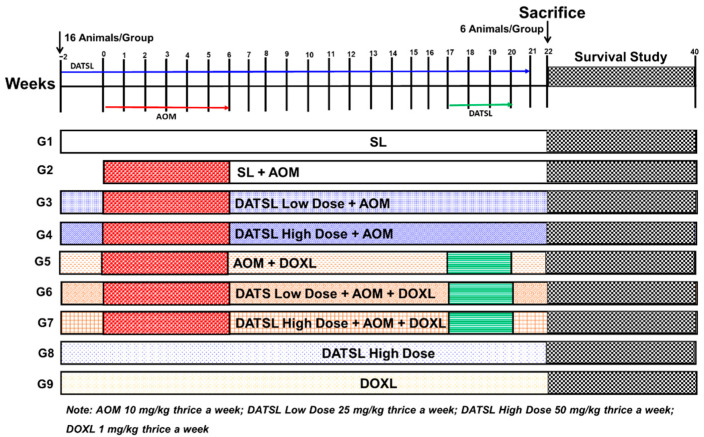
The schematic illustration of experimental design. AOM, G1 (SL) thrice a week from −2 to week 21. G2 mice (10 mg/kg b.w) three times in a week from week 0 to week 6. G3 (low Dose DATSL as 25 mg/kg) three time in a week from week −2 to week 21 + AOM as G2. G4 (High Dose DATSLL as 50 mg/kg) thrice a week from week −2 to week 21 + AOM as G2. G5 (DOXL as 1 mg/kg) three times in a week from week −2 to week 21 + AOM as G2. G6 (Low Dose DATSL as G3 + AOM as G2 + DOXL as G5. G7 (High Dose DATSL as G4 + AOM as G2 + DOXL as G5. G8 (High Dose DATSL as G3 only). G8 (DOXL as G5 only). The mice were given all doses through the intraperitoneal route.

The total 144 mice were randomly divided into nine groups, with 16 mice in each group. The treatment plan for the study is described in the Figure 1. The animals (six mice/group) were sacrificed at the end of 22 weeks after the first exposure of AOM for further biochemical and histological analyses. The survival of rest of the 10 mice in each group was monitored till 40 weeks. The mice were euthanized within 2–4 h when they were moribund, measured by a lack of sustained purposeful response to gentle stimuli during the observational study, and reported dead in survival data.

#### 2.5.3. Assessment of Survival Frequency and Body Weight

The mortality in all the experimental animals was monitored throughout the experimental period till the end of 40 weeks following the first exposure of AOM. The average body weights of mice in each group were recorded at the start of the experiment and continued every 2 weeks for 22 weeks.

#### 2.5.4. Histopathological Analysis of Lung and Liver Tissues

The efficacy of various formulations was assessed on the structural changes induced by AOM in the colon, lungs, and liver cells by histopathological studies of the tissues using H&E staining. Briefly, the formalin-fixed tissues were processed, sectioned, and subjected to hematoxylin and eosin (H&E) staining in a routine manner. The H&E staining of the tissues was studied under the light microscope at 100×, magnifications.

#### 2.5.5. Effect of Various Formulations on AOM Induced Cancer Marker Enzymes in the Serum

The efficacy of various formulations was studied in the serum by determining the activities of carcinogenesis markers as ADA, LDH, γ-GT, and 5′-NT, using Abcam kits as per the instructions given with the respective kits.

#### 2.5.6. Antioxidant Enzyme Assays in Colon Tissues

The effect of various formulations on antioxidant enzymes was investigated as the activities of SOD, CAT, MDA, and GPx1 in the colon of all treated groups of animals. Briefly, the dissected colon tissues were centrifuged in the buffer provided in the kit of the respective enzymes, and the manufacturer’s instructions followed to determine the activity.

### 2.6. Molecular Docking Studies

The Research Collaboratory for Structural Bioinformatics Protein Data Bank (RCSB PDB, http://www.rcsb.org/pdb/home/home.do, accessed on 14 January 2022)) was used to retrieve three-dimensional crystal structures of a number of targets known to be involved in anticancer drug development. Table 2 lists the information of each target, including their PDB IDs and docking predicted binding energies. Biovia Discovery Studio Visualizer 2020 was used to remove co-crystallized water molecules, ligands, and cofactors from each protein. MGLTools 1.5.6 was used to add Gasteiger charges to each receptor, which were then saved in pdbqt format. The diallyl trisulfide and doxorubicin three-dimensional chemical structures were downloaded in sdf format from the PubChem database, then transformed to pdbqt format using MGL Tools 1.5.6 after merging all non-polar hydrogens and defining torsion tree and rotatable bonds. The position of each native co-crystallized ligand in respective proteins was used to allocate binding sites in each target. AutoDock Vina was utilized with the program’s default docking protocol for molecular docking [57]. The optimal postures were chosen from the top models of the ligands in each target by assessing their binding energy (Δ*G*_binding_, kcal/mol) and non-bond interactions profile upon completion of the docking. Biovia Discovery Studio Visualizer 2020, Chimera 1.15, and PyMol 1.7.4 were used to examine molecular interactions as reported previously [58].

### 2.7. Statistical Analysis

The samples in different treated groups were compared by the mean values and standard errors. The significant differences between the treated groups were analysed by One-way and Two-way ANOVA, Tukey’s multiple comparison tests using Prism 9. *p*-value < 0.05 was considered statistically significant.

## 3. Results

### 3.1. Characterization of Liposomes

#### Size, PDI, ζ Potential, and EE

**Figure 2:**
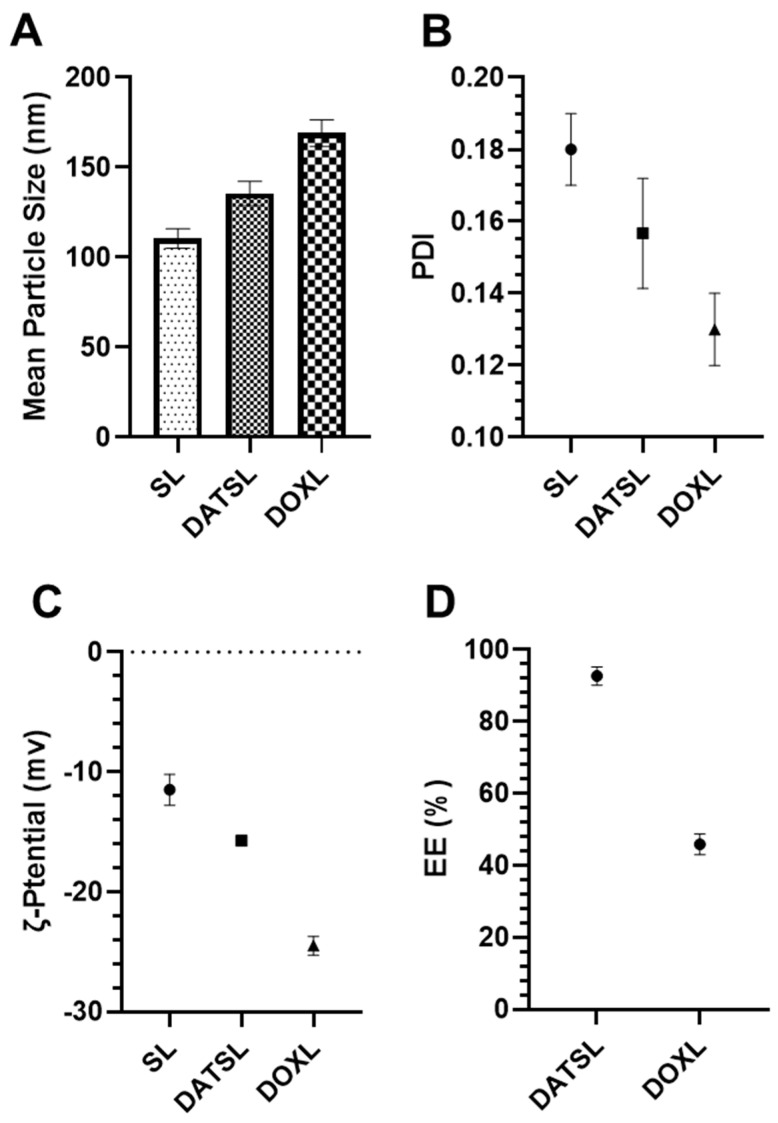
Characterization of DATSL and DOXL. (**A**) Mean particle size. (**B**) Polydispersity index (PDI). (**C**) Zeta (ζ)-potential. (**D**) Entrapment efficiency (EE). The values are expressed as mean ± SE of three independent experiments.

The DLS data revealed that the size of sham liposomes was 110.5 nm with −11.5 mv ζ-potential, while the mean particle size of DATSL and DOXL were scaled to be 135.5 nm and 169 nm, respectively. The ζ-potentials of these liposomes were recorded as −15.75 mv and −24.5 mv, correspondingly (Figure 2A,B). However, all the liposomal formulations showed homogeneity as the <0.2 PDI (Figure 2C). As shown in Figure 2D, the entrapment efficiency was analysed to be ~93% and ~46% in DATSL and DOXL, respectively.

### 3.2. In Vitro Stability of DATS and DOXO Containing Liposomes and Its Release Kinetics

**Figure 3:**
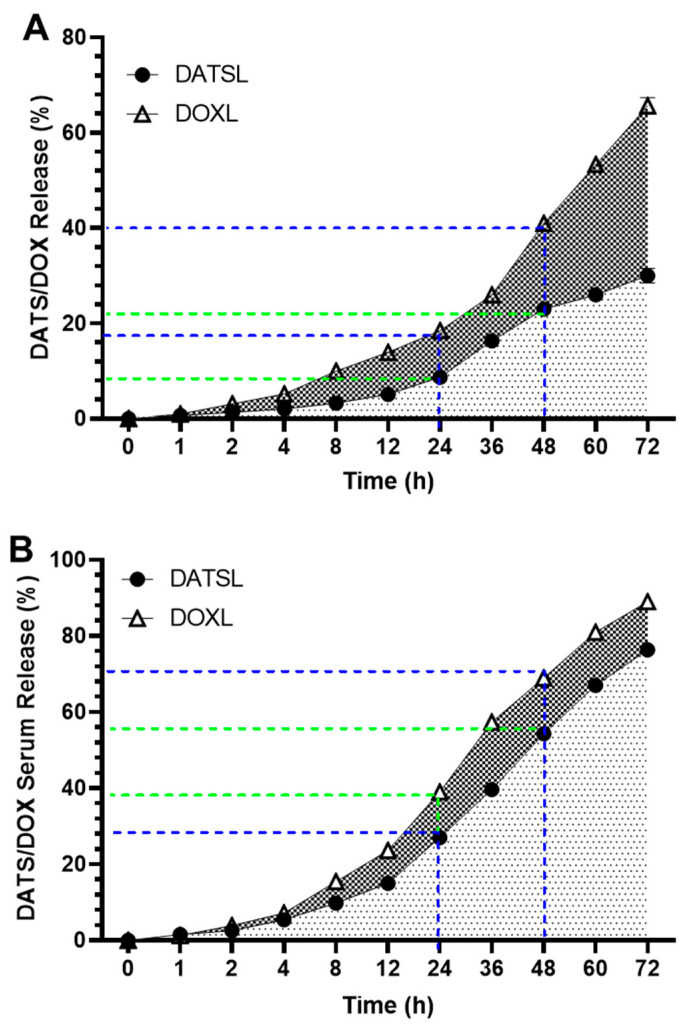
In vitro release of DATSL and DOXL at varying time intervals from 1–72 h. (**A**) Stability of DATSL and DOXL in PBS. (**B**) Release kinetics of DATSL and DOXL in the serum. The values are expressed as mean ± SE of three independent experiments.

The in vitro drug release data showed the stability of DATSL and DOXL, as 30% and 65.7% leakages were measured, respectively, after incubation for 72 h in PBS (Figure 3A). It revealed that only 5% leakage was recorded by DATS and 14% by DOXL after the 12 h. The leakage of DATSL was measured as 8.75%, 16.3%, 23%, and 26% after 24, 36, 48, and 60 h of incubation, respectively. However, the release was calculated as 18.5%, 26%, 41% and 53.4 by DOXL, correspondingly (Figure 3A). The release kinetic results revealed that only 15% and 23.7% were measured in the serum by DATS and DOXL after 12 h, respectively, while it increased to 27% and 39% after 24 h (Figure 3B). The data also demonstrated the continuous but slow release of DATS and DOXO over time, as 39.7%, 54.3% 67%, and 76.3% of DATS estimated after 36, 48, 60, and 72 h, respectively. The release of DOXO was recorded to be 57.3%, 69%, 81%, and 89%, correspondingly (Figure 3B).

### 3.3. Effect of DATS and DOXO on Cellular Proliferation and IC_10_, IC_25_, IC_35_, and IC_50_ at Varying Doses of DATS, DOXO, DATSL, and DOXL in RKO and HT-29 Colon Cancer Cell Lines

**Figure 4:**
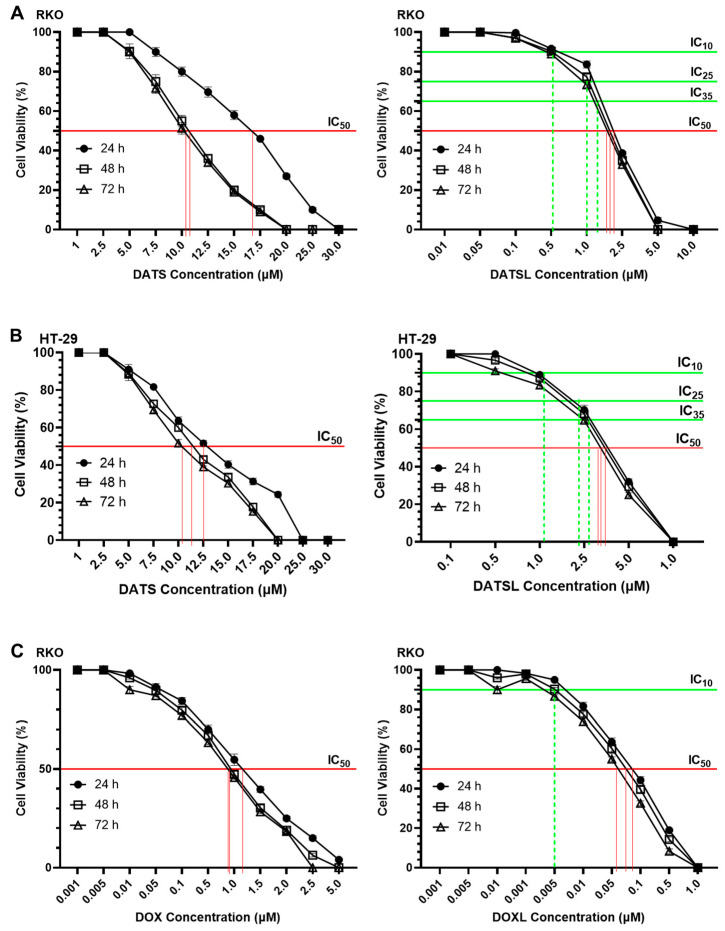

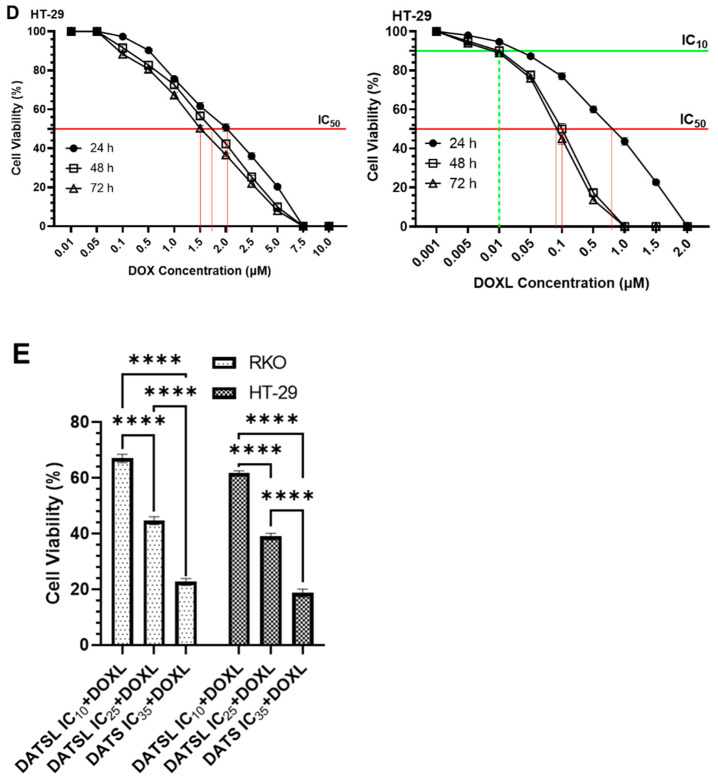
Effect of various formulation on cellular proliferations including IC_10_, IC_25_, IC_35_ and IC_50_ by cell cytotoxicity assay at 24h, 48h and 72h in RKO and HT-29 colorectal cancer cells. (**A**) DATS and DATSL in RKO cells. (**B**) DATS and DATSL in HT-29 cell. (**C**) DOXO and DOXL in RKO cells. (**D**) DOXO and DOXL in HT-29 cells. (**E**) DATSL and DOXL combinations in RKO and HT-29 cells. The values are expressed as mean ± SE of three independent experiments. **** Significant difference between the treated groups, *p*-value < 0.0001.

The cell cytotoxicity data demonstrated the significant difference in the sensitivity of the DATSL and DOXL in comparison to the free forms of DATS and DOXO (Figure 4). The IC_50_ values of DATS were determined to be 16.5 μM, 10.5 μM, and 10.2 μM after 24, 48, and 72 h of the treatment, respectively, while DATSL reduced to 2.0 μM, 1.9 μM, and 1.85 μM, correspondingly, in RKO cells (Figure 3A). The IC_50_ values of DATS and DATSL were recorded as 12.3 (24 h), 11.5 μM (48 h), and 10.5 μM (72 h), and 3.9 μM (24 h), 3.75 μM (48 h), and 3.65 μM (72 h), respectively, in HT-29 cells (Figure 3B). The results showed the IC_50_ of DOXO as 1.1 μM (24 h), 1.0 μM (48 h), and 0.95 μM (72), while it was determined to be 0.09 μM (24 h), 0.08 (48 h), and 0.065 μM (72 h) by DOXL in RKO cells (Figure 4B). The significant differences in the sensitivity of the doses were recorded in both cells in all treated groups after 48 and 72 h, in comparison to 24 h, as the former were found more sensitive. The IC_10_ of DOXL was selected as thw minimal toxic dose for further study to evaluate the chemosensitive potential of DATSL with three different combinations of DATSL: IC_10_, IC_25_, and IC_35_. As the data revealed similar doses of DATSL and DOXL after treating the cells for 48h and 72h, the sensitivity of various combinations was assessed for 48h only. The IC_10_, IC_25_, and IC_35_ of DATSL were calculated as 0.5 μM, 1.0 μM, and 1.4 μM in RKO cells, respectively, while these values were measured as 0.95 μM, 2.1 μM, and 2.6 μM in HT-29 cells, correspondingly (Figure 4A–D). As depicted in Figure 3E, drastic changes were observed in the inhibition of cellular proliferation treated with increasing doses of DATSL in combination with the IC_10_ of DOXL. The results showed 33%, 55%, and 77% inhibition in the RKO cells treated with IC_10_, IC_25_, and IC_35_, respectively, in the combination of DOXL IC_10_, while 48.5%, 61%, and 81.5% inhibition were measured in the HT-29 cells, correspondingly (Figure 4E).

### 3.4. In Vivo Studies

#### 3.4.1. Effect of Various Formulations on AOM Declined Average Body Weight, and Mortality

**Figure 5:**
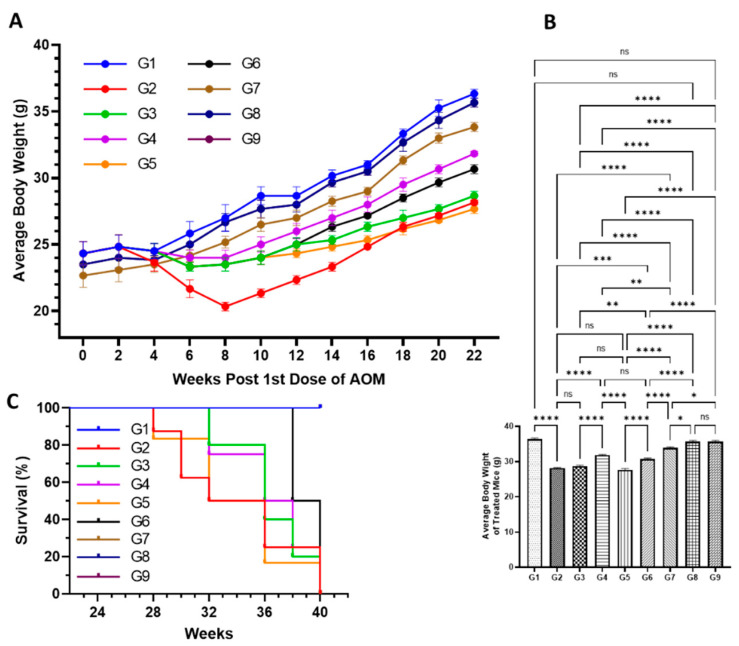
Effect of various formulations on AOM mediate body weight, and mortality. (**A**) Average body weight of the mice during the experiment. (**B**) Average body weight at the end of the experiment before sacrifice. (**C**) Survival rate of animals. ^ns^ No Significance between the treated groups, * Significant difference within the groups, *p*-value < 0.05, ** Significant difference within the groups, *p*-value < 0.01, *** Significant difference within the groups, *p*-value < 0.001, **** Significant difference within the groups, *p*-value < 0.0001.

The results demonstrated significant decrease in the average body weight of the mice exposed to AOM in G2, at 28.2 g, compared to 35.4 g in G1 mice. Interestingly, the high reduction in the bodyweight of G1 mice was noticed during the exposure of AOM from week 4 to week 8, then bodyweight increased from week 10 till week 22, but slowly. However, the significant drop in the average body weight was also observed in all the treated mice from G3 to G7 in comparison to G1, but G4, G6, and G7 mice recovered a significant amount of bodyweight when compared with G2 (Figure 5A,B). The mice which were pretreated with a low dose of DATSL reduced to 28.7 g, while G5 mice that were treated with DOXL decreased to 27.5 g (Figure 5A,B). The mice that were exposed to the combination of DATSL and DOXL showed remarkable retrieval in the body weight, recorded as 34.5 g and 34.6 in G6 and G7 mice, respectively (Figure 5A,B). The Kaplan–Meir curve showed 100% survival in the G7 mice along with G1, G8, and G9, while only 20% mortality was observed in the G6 mice. The data revealed that only 20% survival was found in G2 mice, while it was recorded as 50%, 60%, and 40% in G3, G4, and G5 mice, respectively (Figure 5C).

#### 3.4.2. DATSL and DOXL Reduced the Activities of Cancer Marker Enzymes in the Serum Induced by AOM

**Figure 6:**
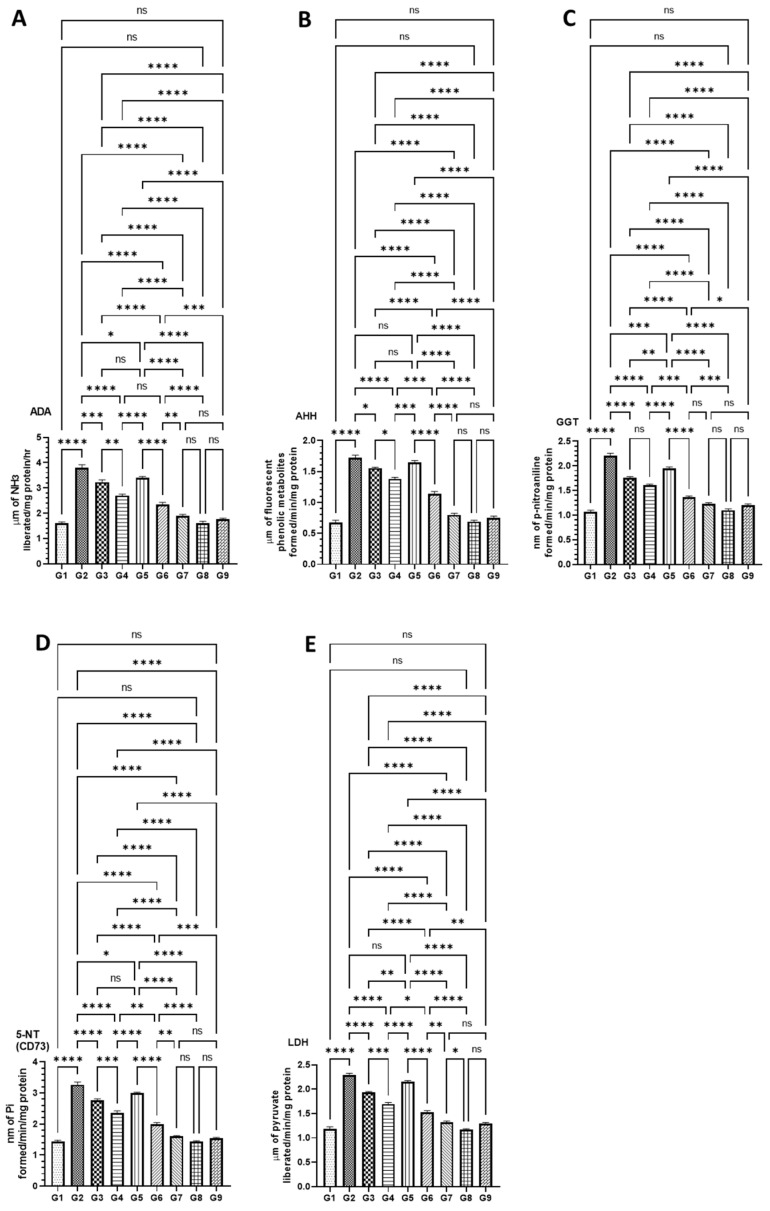
Effect of AOM-induced cancer marker enzymes in the serum (**A**) ADA, (**B**) AHH, (**C**) GGT, (**D**) CD73, and (**E**) LDH. The values are shown as SEM of three independent experiments. ^ns^ No significance between the treated groups, * Significant difference within the groups, *p*-value < 0.05, ** Significant difference within the groups, *p*-value < 0.01, *** Significant difference within the groups, *p*-value < 0.001, **** Significant difference within the groups, *p*-value < 0.0001.

The serum biochemical analyses on the cancer biomarker enzymes studies revealed a significantly strong reduction in the mice treated with a combination of DATSL and DOXL in G6 and G8, which were elevated in G2 mice. A significant drop was also monitored in the mice treated with DATSL and DOXL alone in G3, G4, and G5 (Figure 6). The results demonstrated that the ADA level increased to 3.8 μm in G2, which was measured as 1.6 μm in G1. The synergistic effect could be seen clearly in the G6 and G7 mice as it was recovered to 2.3 μm and 1.9 μm, respectively. The level of ADA dropped significantly in the G3, G4, and G5, at 3.23 μm, 2.7 μm, and 2.7 μm, respectively (Figure 6A). Similarly, the AHH was highly elevated to 1.73 μm in G2 while it was estimated at 0.67 μm in G1. The activities of AH were significantly dropped to 1.55 μm, 1.38 μm, 1.14 μm, and 0.79 μm in G3, G4, G6, and G7, respectively, while no significant recovery was noticed in G5 mice treated with DOXL (Figure 6B). The activities of GGT were significantly increased to 2.2 nm in G2, when compared to G1 as it was calculated to be 1.067 nm. A significant drop was noticed in G6 (1.36 nm) and G7 (1.22 nm), but also in G3 (1.76 nm), G4 (1.61), and G5 (1.95 nm), in comparison to G2 (Figure 6C). Further data demonstrated an upsurge in the activities of CD73 in G2 as it reached 3.26 nm, whereas it was 1.44 nm in G1 mice. The significant fall in the activities of CD73 was noticed in all the treated groups at to be 2.76 nm (G3), 2.35 nm (G4), 3.0 nm (G5), 1.98 nm (G6), and 1.6 nm (G7). Therefore, maximum recovery to the normal level was observed in the G7 mice treated with the combination-2 of high dose DATSL and DOXL (Figure 6D). Similarly, the level of LDH was also found to be increased significantly in G2 mice at 2.28 μm, while it was 1.18 μm in G1. As depicted in Figure 6E, the highest decline to the normal was found in G7 mice at 1.32 μm, followed by G6 (1.52 μm), G4 (1.69 μm), and G3 (1.93 μm), while no significant change was observed in G5 mice. Remarkably, the biochemical analyses of cancer marker enzymes showed no change in the activities of any enzyme in the G8 and G9 mice, when compared to G1 (Figure 6).

#### 3.4.3. Effect of DATSL and DOXL on the Activities of Antioxidant Enzyme in Colon Tissues Modulated by AOM

**Figure 7:**
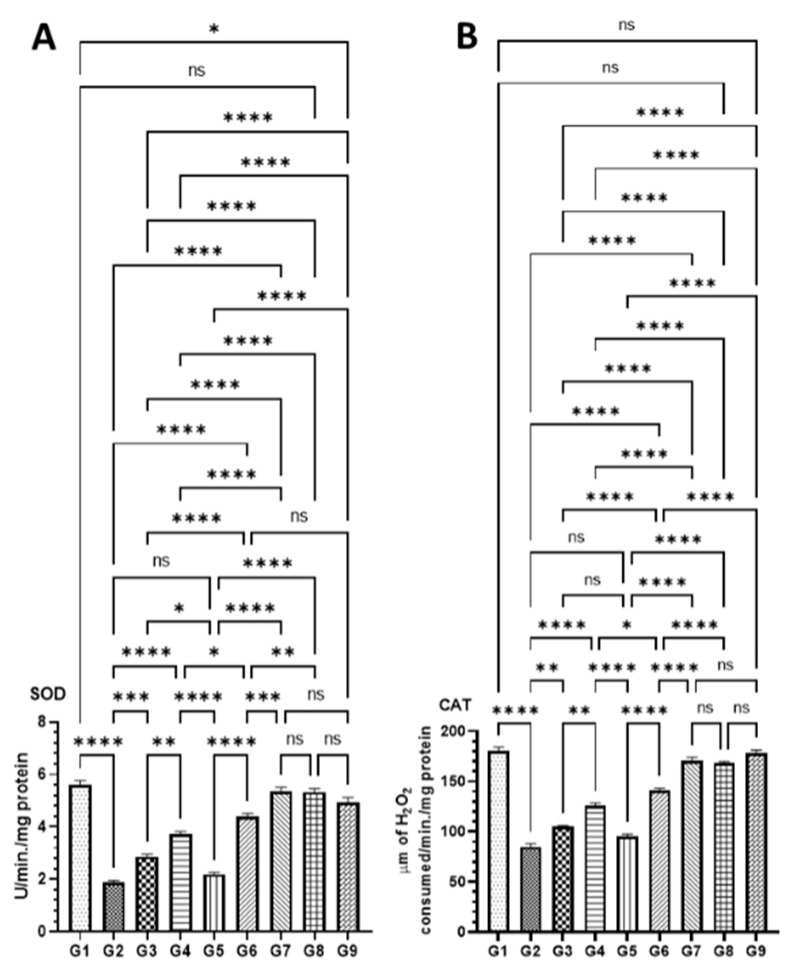

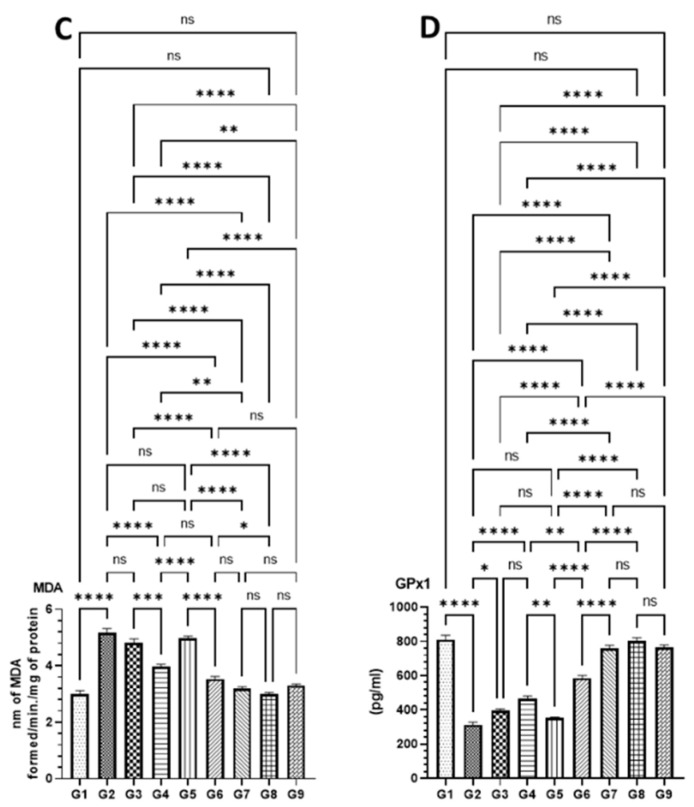
Effect of various formulations on antioxidant enzymes in colon tissues (**A**) SOD, (**B**) CAT, (**C**) MDA, (**D**) GPx1. The values are shown as SEM of three independent experiments. ^ns^ No significant difference between the treated groups, * Significant difference within the groups, *p*-value < 0.05, ** Significant difference within the groups, *p*-value < 0.01, *** Significant difference within the groups, *p*-value < 0.001, **** Significant difference within the groups, *p*-value < 0.0001.

As demonstrated in Figure 7, the level of antioxidant enzymes was significantly affected by AOM as SOD, CAT, and GPx1 activities were dropped, and MDA was increased in G2 mice. However, these changes in the level of antioxidant enzymes were significantly recovered to the normal level in the mice treated high doses of DATSL and DOXL in G7 mice. The data showed that the SOD decreased to 1.9 U in G2, while it was estimated to be 5.6 U in G1. The treatment of DATSL alone or in combination with DOXL made a significant difference in the level of SOD when compared G2, as it increased to 2.86 U (G3), 3.73 U (G4), 4.4 U (G6), and 5.36 (G7). Interestingly, no significant change in the level of SOD was noticed in the animals treated with DOXL alone in G5, when compared to G2 (Figure 7A). The significant drop in the activity of CAT was measured in G2 as 85.0 μm, while it was determined to be 180.33 μm, 168.0 μm, and 178.33 μm in G1, G8, and G9, respectively. The data demonstrated the significant elevation to the normal level of CAT in the G7 was estimated to be 170.66 μm. As exhibited in Figure 7B, the treatment of DOXL alone had no effect on MDA activity in G5, but the DATSL pretreatment in G3 and G4 and combination 1 in G6 showed significant recovery at 105.0 μm, 125.66 μm, and 141.0 μm, respectively. The analysis of MDA activity revealed a significant upsurge in G2 at 5.16 nm, in comparison to 3.0 in G1. However, the treatment of DOXL in G5 could not make a significant change in the activity of MDA induced by AOM, but pretreatment of DATSL significantly reduced to 4.8 nm and 3.96 in G3 and G4 mice, respectively. The treatment of combinations exhibited great reduction, to the normal level of MDA in the G6 and G7 as estimated to be 3.52 nm and 3.2 nm, correspondingly (Figure 7C). Similarly, the GPx1 activity data showed a significant fall in the G2 mice by AOM at 313.33 pg when compared to G1 (810 pg). The results also demonstrated no change in G5 mice treated with DOXL alone, at 355.0 pg. The treatment of DATSL combinations as well as DATSL alone exhibited significant recovery in the level of GPx1 at 296.6 pg, 466.66 pg, 586.66, and 760.0 pg in G3, G4, G6, and G7 mice, respectively (Figure 7D). Remarkably, no significant change in the activity of any activity enzymes was observed in G8 and G9 mice, when compared to G1 (Figure 7).

#### 3.4.4. Effect of DATSL and DOXL on AOM Induced Carcinogenesis in Colon, Lungs, and Liver Tissues by Histopathological Studies

**Figure 8:**
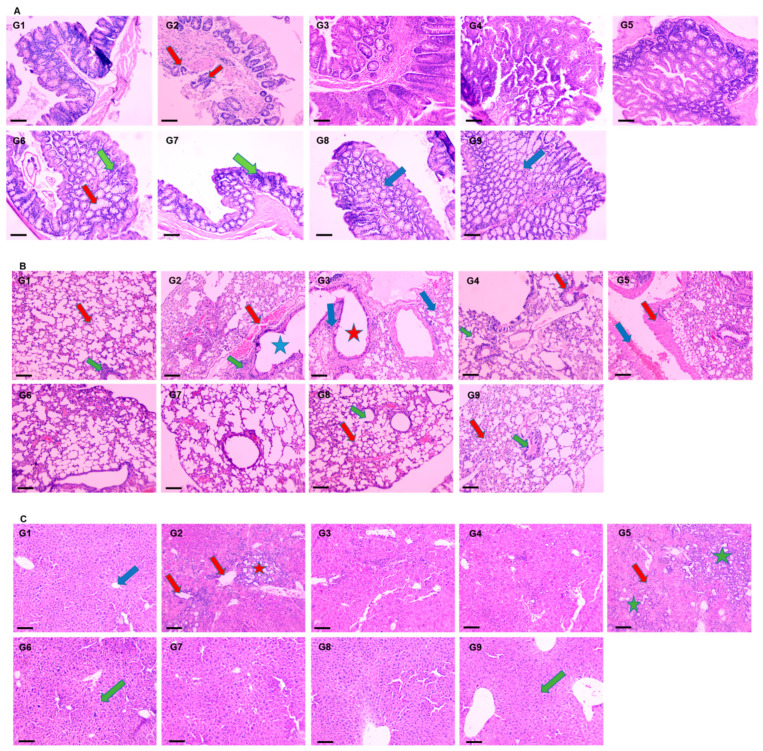
Effect of various formulations on AOM induced carcinogenesis. The representative H&E images of (**A**) colon, (**B**) lungs, (**C**) liver. 100× magnification, bar = 100 µm.

The histopathological results revealed the development of colon cancer with metastasis in the lungs and liver tissues. The mucosal glands were found irregular in size and shape, whereas the acinar glands infiltrated the muscularis mucosae (red arrow) congestion in AOM exposed G2 mice. However, the pretreatment of DATSL showed the inhibition of tumour growth in the G3 and G4 mice, while treatment of DOXL exhibited no effect in tumour growth. The data clearly revealed the high efficacy of DATSL pretreatment with low dose chemotherapy of DOXL in G6 and G7 mice. The submucosal and muscle layer, normal minimal hyperchromatic cells (green arrow) were seen in G6 and G7 mice. However, swelling was observed in the mucosal glands (red arrow) of G6, while none was seen in G7. The normal architecture of mucosal glands, submucosal, and muscle layer was noticed (blue arrow) in G8 and G9 mice, that were given DATSL and DOXL only, as negative controls (Figure 8A). Metastasis was clearly noticed in the lungs of G2 mice, as it showed focal destruction of alveoli separated by thick septa with dilated bronchiole (blue star), high aggregation of leukocytic infiltration (green arrow) with severe haemorrhage (red arrow). However, normal architecture of the alveoli and alveolar sac (red arrow), bronchioles (green arrow) was observed in G1, G8, and G9 mice. The study revealed the moderate destruction of alveoli (blue arrow) with dilated bronchiole (red star), moderate aggregation of leukocytic infiltration and oedema in G3, and minimum destruction of alveoli (green arrow) with dilated bronchiole (red arrow) with less aggregation of inflammatory cells and oedema in G4. The chemotherapy of DOXL in G5 showed focal destruction of alveoli separated by thick septa “fibrosis” (red arrow) with dilated bronchioles, much degeneration, and inflammation with moderate haemorrhage (blue arrow). Remarkably, the DATSL pretreatment with DOXL chemotherapy also showed normal architecture of the alveoli, alveolar sacs, and dilatation of bronchioles with mild leukocytic infiltrations and less congestion as it clearly protected the invasion in the lungs (Figure 8B). The H&E data of liver tissues also showed the invasion and progression of cancer in AOM-exposed G2 mice as an aggregation of tumour cells was arranged in the form of many solid masses (red star), congestion, and severe leukocytic infiltrations in the portal area and central vein (red arrow). Conversely, the G6–G9 mice showed normal central vein and lobular hepatic architecture (green arrow), though mild dilatation of the portal area was noticed in G6. The SL-treated G1 mice exhibited normal hepatic architecture, with normal portal areas of the sinusoids and central vein (blue arrow). As the DOXL-only chemotherapy was not found effective, the H&E staining also showed aggregation of solid masses in and around the portal area and central vein (green star), with moderate inflammations (red arrow) in G5 (Figure 8C).

### 3.5. Molecular Docking Studies

**Figure 9:**
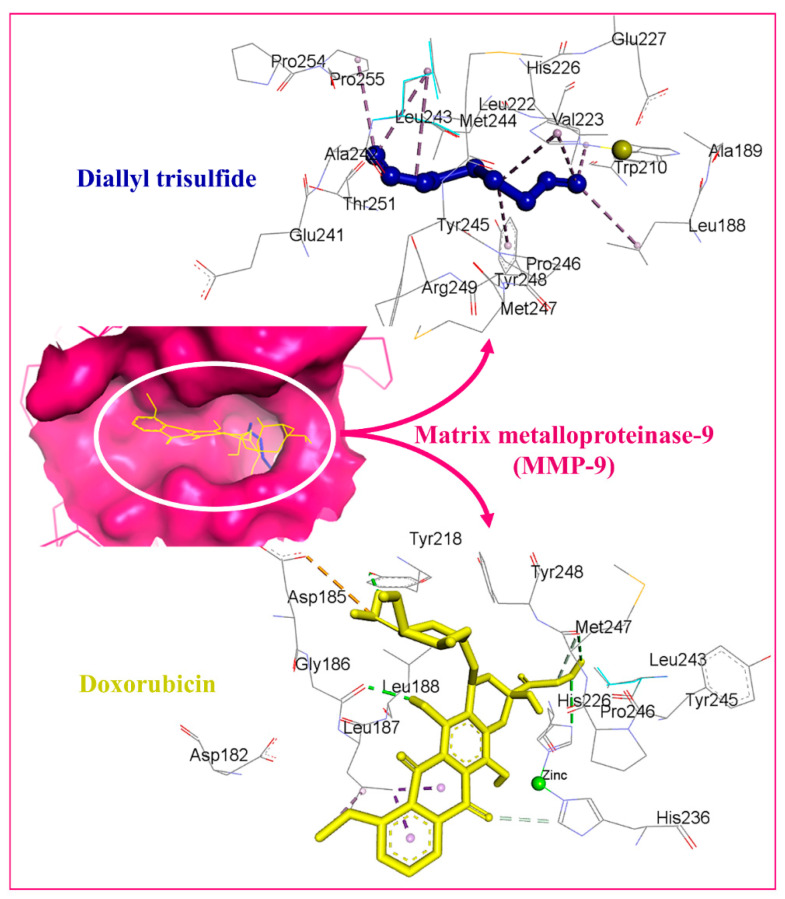
Docked DATS and DOXO, displayed in blue and yellow, respectively. The binding pocket of matrix metalloproteinase-9 is shown as a dark pink surface while interacting residues are shown in grey lines. Intermolecular interactions are represented as dashed lines.

**Figure 10:**
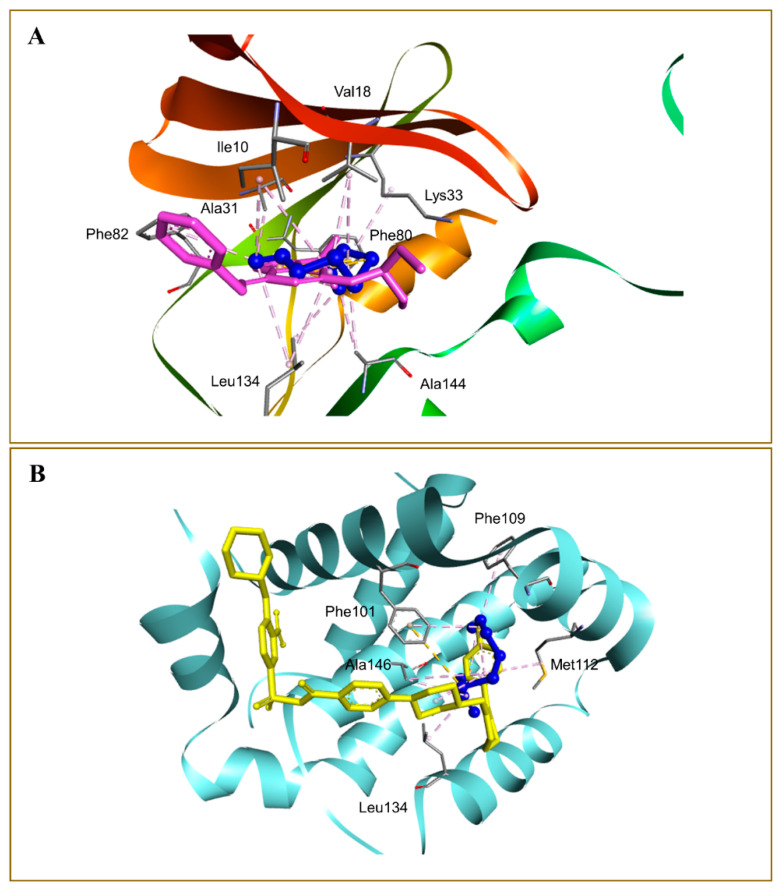
Docked DATS, shown as blue ball-and-stick in the binding pockets of cyclin dependent kinase-2 (**A**), and apoptosis regulator Bcl-2 (**B**) proteins. Native co-crystallized ligands 2-(1-ethyl-2-hydroxyethylamino)-6-benzylamino-9-isopropylpurine (**A**) and 4-(4-{[4-(4-chlorophenyl)-5,6-dihydro-2H-pyran-3-yl]methyl}piperazin-1-yl)-N-{[3-nitro-4-(tetrahydro-2H-pyran-4-ylamino)phenyl]sulfonyl}benzamide (**B**) are shown in purple and yellow sticks, respectively. Non-bond interactions are described as dashed lines.

The binding affinity of the DATS and DOXO against DNA and eleven additional putative anticancer drug targets were predicted using molecular docking (Table 2). The DATS had the highest affinity for MMP-9, with a minimum binding energy of −4.6 kcal/mol, whereas DOXO had a binding energy of −8.9 kcal/mol for this target. DATS exhibited appreciable affinity for the CDK2, apoptosis regulator Bcl-2, and MMP-2 proteins with −4.3, −4.2, and −4.0 kcal/mol, respectively. However, the Janus kinase 2 protein had the highest affinity for DOXO, with −9.6 kcal/mol, whereas DATS had a moderate affinity, demonstrating −3.5 kcal/mol. The data revealed the intermolecular interaction map of docked DATS with CDK2 and the apoptosis regulator Bcl-2 proteins (Figure 9). The docked DATS displayed intermolecular interactions of only the hydrophobic type against all macromolecular targets, whereas both polar and non-polar contacts were established by the DOXO. The interacting residues of MMP-9 include Leu188, Ala189, Trp210, Leu222, Val223, His226, Glu241, Ala242, Leu243, Met244, Tyr245, Pro246, Met247, Tyr248, Arg249, Thr251, Pro254, and Pro255 (Figure 10). Although DOXO is a well-known DNA intercalating agent, moderate binding affinity was exhibited against the nucleic acid in the docking study showing −5.5 kcal/mol. Similarly, the DATS also recorded poor affinity having −2.1 kcal/mol binding energy against the DNA.

## 4. Discussions

The present study demonstrated the chemopreventive and therapeutic potential of liposomal formulations of DATS (DATSL) and DOXO (DOXL) in chemically induced colorectal cancer (CRC) in albino mice. This study is the first to establish the DATSL, and characterized its entrapment efficiency, stability, and release kinetics in the serum. DATSL showed the high EE as ~93% in DATSL with −15.75 mv ζ-potential due to the hydrophobic lipid soluble properties of DATS. The selection of types of phospholipids with cholesterol and their molar ratio with high phase transition (Tc) decides the stability of liposomes [59,60,61,62]. It is not clear the exact proportion, but some researchers have demonstrated the high stability of liposomes in a 2:1 molar ratio of lipid and cholesterol [63,64]. Despite their excellent stability, liposome clearance across RES is the major hurdle in developing novel delivery systems that target extra RES tissues. The inclusion of PEGylated lipids on the upper surface of liposomes improves their circulation time and delivers the entrapped drug/s on to the target site [65,66]. Recently, we showed the high stability with prolonged circulation in the serum by the liposomal formulations of TQ and DADS in the 2:1 molar ratio of DSPC and cholesterol [46,47]. The in vitro release data of DATSL and DOXL demonstrated their great stability and prolonged circulation, as only 30% and 65.6% were measured in the PBS, respectively, at 37 °C after 72 h, while 76% and 89% release were estimated in the serum, correspondingly (Figure 2A). The hydrophobic nature of DATS also might be associated with slow-release as it attaches sturdily to the lipids. There has been no research on the synthesis of DATSL liposomes so far. Therefore, the current study is the first to describe the characterization of DATSL and their in vitro and in vivo pharmacological assessments in CRC models.

Several studies showed that cachexia is one of the important indicators of cancer in chemically induced animal models, which is associated with poor prognosis [67,68,69,70]. The significant drop in body weight was seen in the mice exposed to AOM only, low DATSL with AOM and AOM with DOXL during week 4 to week 8, due to cancer cachexia. Furthermore, the mortality rate was also very high in these groups as recorded as 80%, 50%, and 60%, respectively. However, the treatment of high doses of DATSL and both combinations protected the mice from cancer cachexia conditions, as significant recovery in the body weight, as well as in the survival index, was recorded in G4, G5, and G7 mice after 8 weeks of the experiment (Figure 5).

The increased activities of cancer marker enzymes in the serum are the important biomarkers to diagnose the stage of chemically induced carcinogenesis, as demonstrated earlier [69]. The maximum change in the activities of AHH, γ-GT, 5′-NT, ADA, and LDH to the normal level in the serum by a high dose of DATSL, in combination with DOXL, clearly indicated the former’s role in the delay in cancer initiation, while later inhibited its promotion (Figure 6). Remarkably, the histopathological data ascertained the efficacy of DATSL and DOXL combination as minimal change was observed in the integrity of the colon, while the liver and lung tissues were found intact (Figure 8).

Several antioxidant enzymes play crucial roles in the scavenging of free radicals in ROS-induced oxidative damage. Modulations in the activities of antioxidant enzymes have been shown in colon tissues by colonic carcinogens [71,72,73,74]. As the MDA is the main product of lipid peroxidation, and is considered to have mutagenic and carcinogenic effects, the increased activity of MDA is the sign of an advanced stage of carcinogenesis, its promotion, and progression [69,75]. As depicted in Figure 7, the activities of MDA by AOM in G2 were decreased to the normal level in G7 mice. Our results clearly demonstrate the significant revival of antioxidant enzymes such as GPx1, SOD, and CAT in the mice treated with DATSL and DOXL in G6 and G7, which reduced due to exposure of AOM in G2 mice (Figure 7).

Liposomes are often used to deliver medications for various ailments, including cancer therapy. The formulation’s active ingredient, on the other hand, has pharmacological effects only when it interacts with enzymes, receptors, signal transduction proteins, and other targets. In this context, the molecular docking approach is commonly used to determine how medicinal compounds of liposomal formulations interact with the macromolecular targets [76,77]. Therefore, molecular docking analysis was performed to determine DATS’s ability to interact with prospective anticancer therapeutic targets. The technique was used for the first time to define the molecular interactions profile of DATS against a variety of drug-targets important to carcinogenesis. Doxorubicin, used as a standard drug in this study, was also included in the molecular docking analysis. The maximum affinity of DATS was revealed against MMP-9 in a docking experiment followed by cyclin dependent kinase-2 (CDK2), apoptosis regulator Bcl-2, and MMP-2 proteins. Interestingly, DATS has been reported for inhibiting the migration and invasion of 5637 cells by decreasing the activity and expression of MMP-2 and MMP-9 at both the protein and mRNA levels in an experimental finding by Shin and co-workers [78]. MMP-2 and MMP-9 are gelatinases that break down collagens and laminins. Both MMP-2 and MMP-9 have been linked to tumour growth, cell death, inflammation, bone remodelling, and immunity [79]. The expression of these enzymes has been found to be high in numerous malignant cells, making it a prognostic factor and a viable target for anticancer treatment [80]. These investigations indicate that DATS and DOXO possesses an appropriate molecular arrangement to interact with several pharmacological targets that may correlate with their anticancer activity. However, changes in chemical structure may be attributable to their differences in docking-predicted binding energies. Despite molecular docking being a powerful computational technique for determining the strength of intermolecular interactions between ligands and proteins, the method fails to account for the solvation penalty associated with binding necessitating further investigation to authenticate the predicted binding energies. These results might serve as a basis for further investigation into the DATS’s unique anticancer mode of action.

## 5. Conclusions

In the present study, we developed the PEG coated long circulating, liposomes of DATS and DOXO. The results summarize the chemopreventive and chemosensitive profile of DATSL in an animal model of AOM induced colorectal cancer. The pre-treatment of DATSL with DOXL was not only gave protection from liver and lung metastasis, it also prevented the promotion of carcinogenesis in the colon. This is the first study in the development of DATS formulations, considering its limited applicability in clinical settings due to poor solubility. In order to fully comprehend the molecular mechanism of DATSL and DOXL in the treatment of colon cancer, more research is needed, concentrating on the molecular targets of DATS and DOXO suggested by molecular docking studies.

## Figures and Tables

**Table 1:** The range of the concentration of DATS, DOXO, and their formulations for cell cytotoxicity assay in RKO and HT-29 CRC cell lines.

Colorectal Cancer Cells	Formulations			
	DATS	DATSL	DOXO	DOXL
RKO	1–30.0 μM	0.01–10.0 μM	0.001–5.0 μM	0.001–1.0 μM
HT-29	1–30.0 μM	0.1–1.0 μM	0.01–10.0 μM	0.001–2.0 μM

**Table 2:** AutoDock Vina results of diallyl trisulfide and doxorubicin against potential anticancer drug targets.

S.N	Targets	PDB Code	Binding Energy (kcal/mol)	
			Diallyl Trisulfide	Doxorubicin
1	Matrix metalloproteinase-9 (MMP-9)	5CUH	−4.6	−8.9
2	Cyclin dependent kinase 2 (CDK2)	2A4L	−4.3	−9.3
3	Apoptosis regulator Bcl-2	4LXD	−4.2	−7.2
4	Matrix metalloproteinase-2 (MMP-2)	1HOV	−4.0	−7.8
5	Nrf2-Keap1	4L7D	−3.6	−8.9
6	Phosphatidylinositol-3 kinase alpha (PI3K-α)	3ZIM	−3.6	−8.9
7	Pyruvate dehydrogenase kinase (PDK)	4V26	−3.5	−7.5
8	Janus kinase 2 (JAK2)	3KCK	−3.5	−9.6
9	Tubulin beta	1JFF	−3.3	−8.1
10	TNF-alpha	3WD5	−3.3	−6.8
11	Nuclear factor kappa-B (NF-KB)	1NFK	−2.8	−5.9
12	DNA	1P20	−2.1	−5.5

## Data Availability

Not applicable.
